# Bioavailability of arsenic, cadmium, lead and mercury as measured by intestinal permeability

**DOI:** 10.1038/s41598-021-94174-9

**Published:** 2021-07-19

**Authors:** Shiv Bolan, Balaji Seshadri, Simon Keely, Anitha Kunhikrishnan, Jessica Bruce, Ian Grainge, Nicholas J. Talley, Ravi Naidu

**Affiliations:** 1 Global Centre for Environmental Remediation, University of Newcastle, Callaghan, NSW Australia; 2 Cooperative Research Centre for Contamination Assessment and Remediation of the Environment, University of Newcastle, Callaghan, NSW Australia; 3 Hunter Medical Research Institute, New Lambton Heights, NSW Australia; 4 School of Environmental and Life Sciences, University of Newcastle, Callaghan, NSW Australia

**Keywords:** Biological techniques, Cell biology, Microbiology, Environmental sciences, Gastroenterology, Medical research, Chemistry

## Abstract

In this study, the intestinal permeability of metal(loid)s (MLs) such as arsenic (As), cadmium (Cd), lead (Pb) and mercury (Hg) was examined, as influenced by gut microbes and chelating agents using an in vitro gastrointestinal/Caco-2 cell intestinal epithelium model. The results showed that in the presence of gut microbes or chelating agents, there was a significant decrease in the permeability of MLs (As-7.5%, Cd-6.3%, Pb-7.9% and Hg-8.2%) as measured by apparent permeability coefficient value (*P*_app_), with differences in ML retention and complexation amongst the chelants and the gut microbes. The decrease in ML permeability varied amongst the MLs. Chelating agents reduce intestinal absorption of MLs by forming complexes thereby making them less permeable. In the case of gut bacteria, the decrease in the intestinal permeability of MLs may be associated to a direct protection of the intestinal barrier against the MLs or indirect intestinal ML sequestration by the gut bacteria through adsorption on bacterial surface. Thus, both gut microbes and chelating agents can be used to decrease the intestinal permeability of MLs, thereby mitigating their toxicity.

## Introduction

Non-essential heavy metal(loid)s (MLs) such as arsenic (As), cadmium (Cd), lead (Pb) and mercury (Hg) have been associated with human health risks. Through various exposure pathways, these MLs can become bioavailable and lead to toxicity and poisoning^1^. Bioavailability is determined by the ability of a compound to start circulating in a living system after being absorbed by the intestine, which can be determined using in vivo or in vitro assays^1,2^. Several in vitro cell-line-based (e.g., Caco-2, Human colorectal adenocarcinoma Tumour cell line with epithelial morphology (HT-29), and Madin-Darby canine kidney (MDCK)) or tissue-based (e.g., Everted intestinal ring) systems, and artificial membrane (e.g., Parallel artificial membrane permeability assay (PAMPA)) techniques are some of the methods used to evaluate the possible permeability of nutrients, drug compounds and MLs in the intestine^3–6^. Through oral ingestion, the nutrients and contaminants are stopped from entering the circulatory system by intestinal epithelial cells, which serves as an initial barrier. Several researchers used Caco-2 cells to study absorption mechanisms and to evaluate the permeability of drugs, nutrients, and minerals through the intestinal cells^3,7,8^. The in vitro bioavailability study is usually carried out through assessing the concentration of compounds present in simulated gastrointestinal media and their bioaccessibility^1^. This approach of measuring bioavailability can be improved using Caco-2 cell model, which mimics the process of intestinal cell retention and transport^5,9^.

The human colon adenocarcinoma cells have the ability to segregate into single layers of polarised enterocytes, which can be cultured and established into Caco-2 intestinal cell line^5,8^. The segregated single layer of cells is polarised, with microvilli on the apical border, enzyme secretion characteristic to the brush border membrane, intercellular tight junctions (TJ), and the expression of transporters typical to the small intestine in the apical and basolateral membranes^10,11^. Caco-2 cell line has been predominantly used in research pertaining to nutrient and drug absorption^12,13^. Nowadays, this cell line is used to assess the effect of environmental contaminants on intestinal permeability and the resultant absorption^14–16^. Several researchers have validated the transportation of drug compounds through Caco-2 monolayer by assessing in vivo absorption in human intestine^5,8,13^.

The ability of a ML ion to pass through the gastrointestinal barrier is a key property to consider when examining the bioavailability and toxicity of ingested heavy MLs^17–19^. The mechanisms of ML permeation through biological barriers include passive diffusion (or paracellular) and active (or transcellular) transport pathways^19–21^. Passive diffusion of MLs is a physicochemical process that depends on properties such as lipophilicity, hydrogen bonding, stability constant (pKa) of the ML complex, molecular weight and test conditions, for example, the pH gradient and permeation time. In passive, paracellular absorption, the ML ions diffuse through tight junctions (TJ) into the basolateral spaces around enterocytes, and hence into blood^22,23^. Active, transcellular absorption involves import of MLs into the enterocyte, transport across the cell, and export into extracellular fluid and blood. Active transport involves active carrier mediated transportation and the use of energy to transport specific substrates across barriers, even against the concentration gradient^20^.

Intestinal absorption could be amplified after chronic ML exposures. For instance, cell death after chronic Cd exposure may cause leakage in the epithelial layer, resulting in larger amounts of Cd permeation^16^. Furthermore, Cd-induced disruption of TJs may lead to an intercellular leakage, allowing Cd to pass through the intestinal barrier^16^. Tight junctions are located in the apical part of the intestinal epithelial cells and are composed of a large group of proteins, including the scaffolding proteins zonula occludens-1 (ZO-1), and the transmembrane proteins, occludin and claudins, which are crucial in maintaining the barrier function^22,24,25^. When the expression of the TJ proteins is altered, the functionality of this physical barrier is compromised^22^ and may lead to a leaky gut which is characterised by having an epithelium with increased permeability to compounds that diffuse from the lumen to the lamina propria^24,26,27^.

In this paper, the effect of gut bacteria and chelating agents on the bioavailability of heavy MLs as measured an in vitro model for intestinal permeability will be reported. The process of intestinal absorption of heavy MLs may be affected by their binding with compounds like chelating agents that reduce their passage through the epithelium, and also with their binding or interaction with gut microorganisms^28,29^. The gut microbes provide benefits to the host gut and prevent intestinal barrier dysfunction by: (i) modulating immune responses, (ii) alleviating oxidative stress (iii) reducing intestinal permeability by maintaining intestinal barrier integrity through expression and distribution of TJ proteins, and (iv) inhibiting abnormal necrosis of epithelial cells^26,27,30^. Chelating agents such as ethylenediaminetetraacetic acid (EDTA), 2,3-dimercapto-1-propanesulfonic acid (DMPS) and dimercaptosuccinic acid (DMSA) have been shown to increase ML bioaccessibility, thereby influencing the absorption and bioavailability of MLs in the intestine. While the Caco-2 cell technique involving intestinal epithelial cell monolayers has been widely used to study drug and nutrient absorption, it has been less used to understand the intestinal permeability of MLs in the presence of gut microbes and chelating agents, which is the main focus of this paper.

The overall objective of this work reported in this paper was to examine the bioavailability of orally ingested As, Cd, Hg and Pb as measured by intestinal permeability using a Caco-2 cell model. The specific objectives in this paper were to:

(i) Evaluate intestinal permeability of As, Cd, Hg and Pb in the presence of intestinal extract.
(ii) Examine the effect of gut microbes (*Escherichia coli* and *Lactobacillus acidophilus*) on the intestinal permeability of As, Cd, Hg and Pb in the presence of intestinal extract.
(iii) Investigate the impact of chelating agents (EDTA and DMPS) on the intestinal permeability of As, Cd, Hg and Pb in the presence of intestinal extract.

The hypotheses tested include;

(i) Bioavailability of heavy MLs as measured by intestinal permeability is impacted by ML binding with compounds or gut microbes that reduce their solubility (i.e., bioaccessibility) or their passage through the epithelium.
(ii) Gut bacteria modulate bioaccessibility of MLs as measured by intestinal permeability through their interactions with MLs via adsorption and chemical speciation processes.
(iii) Chelating agents alter the bioaccessibility of MLs by forming complexes with MLs, thereby influencing the intestinal absorption of MLs.

## Materials and methods

### Metal(loid) sources

The ML sources included in this study are arsenic oxide (As III), cadmium acetate (Cd), lead acetate (Pb), mercuric chloride (Hg). These ML sources were selected because these are readily soluble and have often been used for in vivo ML bioaccessibility assessment^31,32^, and also in toxicity studies in the Integrated Risk Information System^33^.

### Gut microbes and Chelating agents

*Escherichia coli* and *Lactobacillus acidophilus* were selected as gut microbes to study their effect on heavy ML bioavailability in the presence of intestinal extracts as measured by intestinal permeability test using Caco-2 cells. It is important to recognise that the human gut microbiota are a composite structure of a large number of distinct bacterial species that reside in the human digestive tract. In this study, these two bacterial species were used based on their cell wall structure (gram positive and gram negative), predominance in the gut, differences in their pH optimum in the gut and their location in various parts of the human gut (Supplementary Table 1). Subcultures of these bacteria were inoculated from their respective mother cultures purchased from American Type Culture Collection (ATCC, Melbourne; https://www.atcc.org/). The growth of the two bacterial species was studied in the presence of various concentrations (0, 0.1, 1.0, 5.0 and 10 mmol/L) of the two chelate solutions (EDTA and DMPS). The bacterial species were inoculated in the growing media containing the chelating agents and monitored over a period of 24 h in a 96-well round bottom microplate (Costar 3799, CORNING INCORPORATED, USA) under sterile anaerobic conditions at 37 °C. The bacterial growth was monitored by measuring optical density (@600 nm) over time in a microplate reader (BMG LABTECH FLUOstar OPTIMA Fluorescence Microplate Reader, Germany).

The chelating compounds included in this study are based on their potential applications in the treatment of heavy ML toxicity. The most common synthetic chelating agents used to manage acute ML poisoning in humans are ethylenediaminetetraacetic acid (EDTA), 2,3-dimercaptopropane-1-sulfonate (DMPS), 2,3-dimercaptosuccinic acid (DMSA), 2,3-dimercatopropanol (BAL), Deferoxamine, Deferiprone and Deferasirox. In this study, EDTA and DMPS (both chelates at 1 mM concentration) which are used commonly to treat ML toxicity were selected to study their influence on heavy ML bioavailability in the presence of intestinal extracts as measured by intestinal permeability test using Caco-2 cells (Supplementary Table 2).

### Cell culture

**Figure 1:**
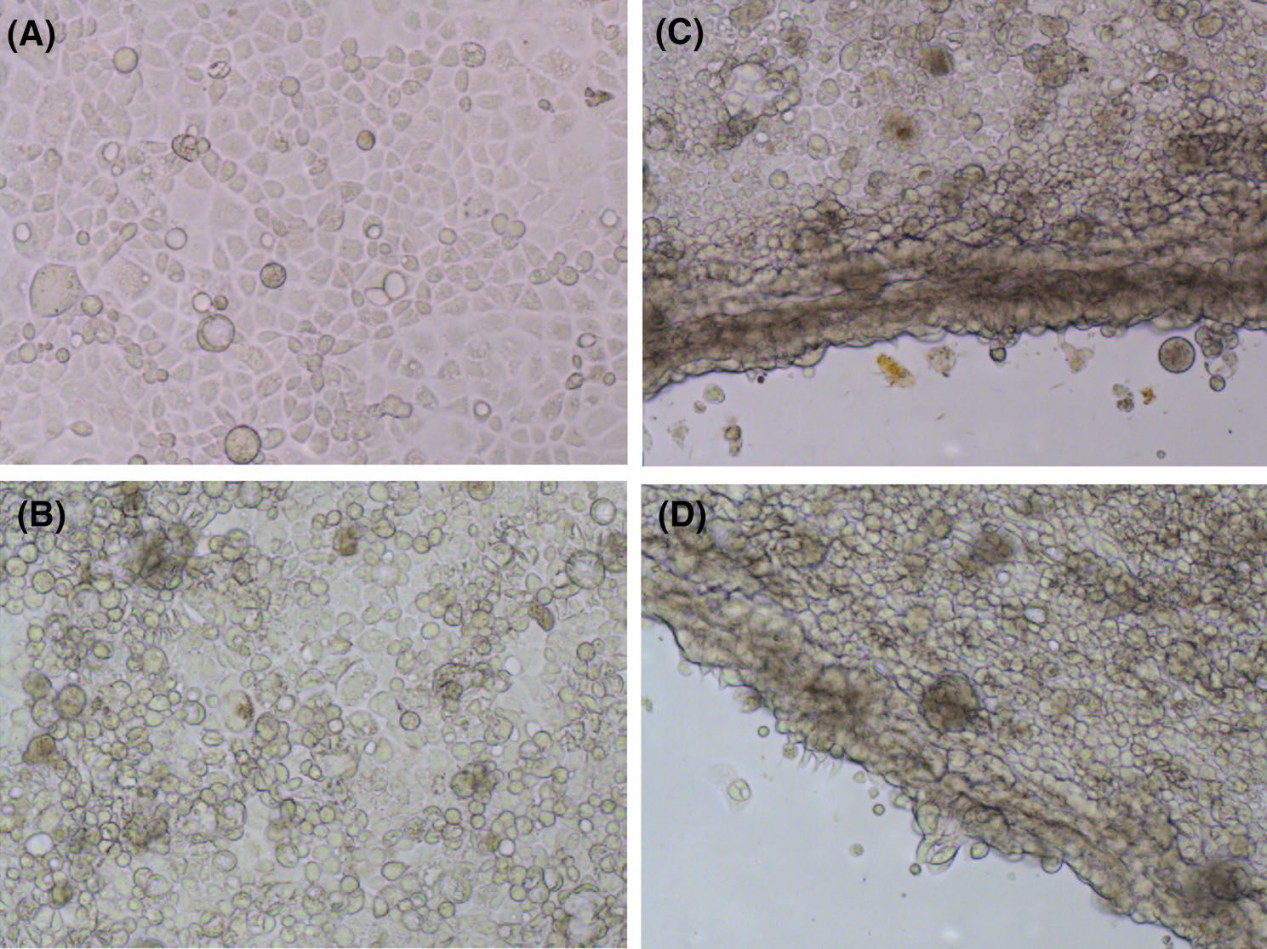
Caco-2 epithelial cell organisation. Cells were cultured on permeable membrane filter support for 20 days. Healthy cells (**A**) and their attachment to the membrane plate (**B**) in the presence of reference metal(loid) samples. Damaged cells (**C**) and their attachment to the membrane plate (**D**) in the presence of metal(loid) source samples used in bioaccessibility tests. Hence only reference metal(loid) samples were used for the bioavailability tests as measured by intestinal permeability using Caco-2 cell technique.

The Caco-2 cells were acquired from Hunter Medical Research Institute, University of Newcastle, Australia. The Caco-2 cells were routinely grown in 75 cm^2^ flasks in Dulbecco's modified Eagle's medium (DMEM) at pH 7.4 containing glucose (4.5 g/L) and L-glutamine (0.6 g/L) and supplemented with 10% (v/v) fetal bovine serum (FBS), 1% (v/v) non-essential amino acids, 10 mM HEPES (N-2-hydroxyethylpiperazine-N′-2-ethanosulfonic acid), 100 U/mL of penicillin, 0.1 mg/mL of streptomycin, 0.0025 mg/mL of fungizone, and 1 mM sodium pyruvate^13,34^. The cell lines were incubated at 37 °C, in a humidified atmosphere of 95% air and 5% CO_2_, and the medium was changed every 2−3 days. When the cell monolayer reached 80% confluence, the cells were detached with a solution of trypsin (0.5 mg/L) followed by reseeding at a density of 0.5 × 10^6^ cells/cm^2^ (Fig. 1).

### Cell retention, transport, and permeability tests

The cell retention, transport, and permeability tests were performed in two chamber wells with polyester membranes (diameter 24 mm; pore size 0.4 μm; Transwell, COSTAR CORPORATION, NY)^34^. In this system, the cells were kept on a porous support that separates the well into two compartments: apical and basal (or basolateral) chamber wells. The Caco-2 cells were seeded at 0.5 × 10^6^ cells/cm^2^. The media, Dulbecco's Modified Eagle's medium (DMEM) with 5% FBS (0.5 mL in apical and 1.5 mL in basolateral compartments), was changed every 2 days until cell differentiation was achieved, with mature Caco-2 cells obtained after 17–19 d post seeding^35^. ML permeability/transport test was performed 21 days post seeding of Caco-2 cells. The filter insert (i.e. apical chamber) was rinsed with DMEM (without Phenol Red) pH 7.2 supplemented with 10 mM HEPES (4-(2-hydroxyethyl)-1-piperazineethanesulfonic acid) buffer and 15 mM L-glutamine, and allowed to equilibrate at 37 °C for 15 min in the incubator. The test solutions contained 2.5 mg/mL Fluorescein isothiocyanate (FITC)-dextran (Mw 4400) (FD-4) as a paracellular marker. The test solutions also contained gut bacteria (*E. coli* and *L. acidophilus*) and chelating agents (1 mM EDTA and DMPS). For the uptake (retention and transport) assay with cells, the intestinal extract of MLs was heated for 4 min at 100 °C to inhibit sample proteases and then cooled by immersion in an ice bath.

The permeability tests were initiated by replacing the apical (0.5 mL) buffer with the test intestinal extract ML solutions. The test solutions were diluted with DMEM medium (1:3) before adding to the apical compartment. To diminish the unstirred water layer, transport experiments were carried out under agitation (70 Hz) in a plate shaker maintained at 37 °C. A 500 μL sample was collected from the basolateral (1.5 mL) chamber at every 20 min and replaced with fresh buffer. Sampling of basolateral solution was continued for 120 min period. At the end of the assay, the cells were recovered by washing in phosphate buffered solution (PBS), scraped, and then lysed with 1% Triton X-100 (Merck, Germany). The MLs in the basolateral compartment and in the cells were quantified. The cell surfaces of the monolayers were washed three times with PBS, detached with a trypsin solution, and recovered with 0.5 mL of PBS^36^. The ML retention and transport percentages were calculated with respect to the initial quantity of ML added to the Caco-2 cell cultures. The respective samples were analysed for ML concentrations using ICP-MS.

### The distribution of free and complexed metal(loid)s

The distribution of free and complexed MLs in the gastric and intestinal extracts was measured using chelate exchange disk/ cation-exchange resin cartridge (Empore, iminodiacetate functionalized poly(styrene divinylbenzene)—234877 Aldrich)^37^. Exactly 5 mL of 3.0 M nitric acid and 5 mL of Milli-Q water were sequentially passed through the cartridge. Then, 3 mL of the gastric or intestinal extract was passed through the cartridge, and 5 mL of Milli-Q water was passed through to rinse the cartridge. The 8 mL of leachate was collected and determined for MLs using ICP MS. Free ionic forms of MLs are retained in the cation-exchange resin cartridge. The ML concentration in the leachate solution is considered to be stable complexed MLs, and the difference between total concentration and complexed MLs concentration measured in the filtrate gives the ionic free MLs concentration. The distribution of As(V) and As(III) species was measured using HPLC-ICP-MS hyphenated set-up^38^. A system of liquid chromatography hyphenated to an inductively coupled plasma mass spectrometer (HPLC-ICP-MS) from PERKIN ELMER (Sunnyvale, CA, USA) was used, consisting of a P680 HPLC pump, an ASI-1 00 automated sample injector and an Elan DRC-e ICP-MS detector (PERKIN ELMER, Sunnyvale, CA, USA).

### Data analysis

The apparent or absolute permeability coefficient (*P*_app_ = cm/s) can be calculated from concentration–time profiles using the following equation^39^:

1 $$P_{{{\text{app}}}} \,{\text{(cm}}/{\text{s}}) = {\text{dC}}/{\text{dt}}*1/{\text{A}}*{\text{V}}/{\text{C}}_{{\text{o}}}$$

where, dC/dt (µg/mL/s) represents the flux across the monolayer (ML concentration (µg/mL) at various time (t in seconds) period); A (cm^2^) the surface area of the monolayer; V (cm^3^) the volume of the receiver chamber; and C_o_ (µg/mL) the initial ML concentration in the donor compartment.

The relative permeability values (*P*_rel_) were estimated using (Eq. 2) to examine the effect of various treatments (gut microbes and chelating agents) on intestinal apparent permeability values (*P*_app_).

2 $$P_{{{\text{rel}}}} \,(\% ) = (P_{{{\text{app}}}} {\text{treatment}}/P_{{{\text{app}}}} {\text{Control}})*100$$

where *P*_app_ treatment is apparent permeability value for the test solution with a particular treatment (gut microbe or chelate addition) and *P*_app_ Control is apparent permeability value for the control treatment.

All the experimental analyses were carried out using three replications. The permeability tests were conducted using Caco-2 cells grown for three passages. The passage number of a cell culture is a record of the number of times the culture has been subcultured, i.e. harvested and reseeded into multiple ‘daughter’ cell culture flasks^40^.

Statistical comparisons were made using analysis of variance (ANOVA) in Predictive Analytics SoftWare (PASW) statistics (version 18.0.0; SPSS, Inc., 2009, Chicago, IL) in order to examine the significant differences in various treatments. Duncan's multiple range test was also employed to compare the means of various treatments; variability in the data was presented as the standard deviation and a *p* < 0.05 was considered statistically significant.

## Results

### Transport of metal(loid)s and apparent permeability

**Figure 2:**
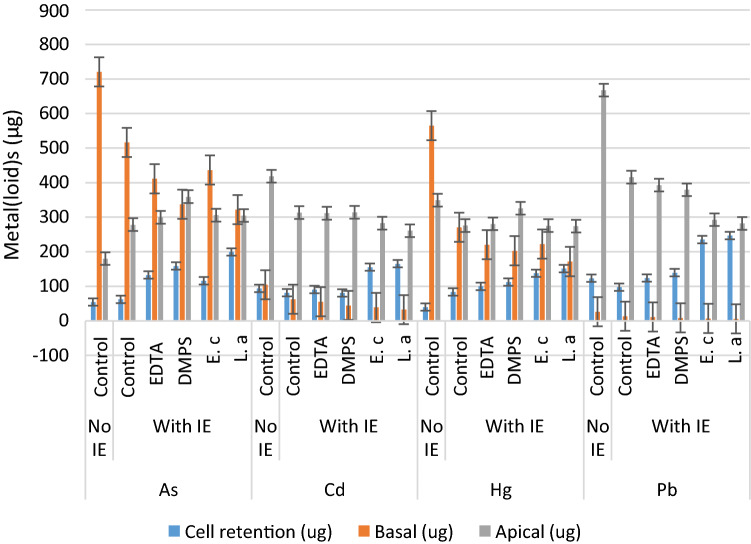
Distribution of metal(loid)s in the apical chamber, basolateral chamber and retention by cells during permeability test using Caco-2 cell technique. IE, intestinal extract; EDTA, ethylenediaminetetraacetic acid; DMPS, 2,3-dimercaptopropane-1-sulfonate; ; *E.c*, *Escherichia coli*; *L.a*, *Lactobacillus acidophilus.*

The transport of MLs in the direction of apical to basolateral compartment of Caco-2 monolayer was assessed. Mass balance calculations were carried out to estimate the distribution of MLs in the basolateral well (permeable fraction), apical well, and Caco-2 cells (cell retention) (Tables 1 and 2; Fig. 2). The mass balance indicated that the total recovery of ML in the Caco-2 technique ranged from 89.7 to 105.3%, and there was a slight decrease in the recovery in the presence of gut bacteria. The total uptake values (cell retention plus basolateral transferred) of As, Cd, Pb and Hg in the Caco-2 cells were 81.9%, 32.9, 65.6% and 18.9%, respectively, in the absence of intestinal extract and 67.3%, 17.3%, 61.2% and 3.45%, respectively, in the presence of intestinal extract (Table 2) indicating that the intestinal extract decreased the uptake of MLs.

**Table 1:** Mass balance of metal(loid)s during permeability test using Caco-2 cell technique.

Metal(loid) sources*	Chelating agents/Gut Bacteria	Metal(loid)				
		Total input (µg)**	Apical (µg)	Membrane retention (µg)	Basolateral (µg)	Total (% of input)
As—No IE	Control	945	180	54.2	720	101.1
As—with IE	Control	860	278	62.7	516	99.6
	EDTA	868	300	133	411	96.7
	DMPS	865	359	159	337	98.7
	*E. coli*	872	306	116	437	98.2
	*L. acidophilus*	856	305	199	322	95.4
Cd—No IE	Control	606	419	94.5	105	102.2
Cd—with IE	Control	460	313	81.2	62.6	99.2
	EDTA	472	311	91.4	55.2	96.2
	DMPS	465	314	81.8	45.5	93.4
	*E. coli*	481	283	155	39.6	98.7
	*L. acidophilus*	470	261	165	32.4	96.3
Hg—No IE	Control	923	349	40.6	565	103.5
Hg—with IE	Control	635	276	84.5	271	99.1
	EDTA	647	281	100	221	91.6
	DMPS	653	326	112	203	97.8
	*E. coli*	640	275	138	222	99.1
	*L. acidophilus*	648	274	151	172	89.7
Pb—No IE	Control	796	668	123	27.5	103.2
Pb—with IE	Control	530	416	98.2	14.5	99.3
	EDTA	553	393	124	11.3	94.2
	DMPS	546	379	140	9.35	95.6
	*E. coli*	538	293	235	7.56	99.0
	*L. acidophilus*	546	282	247	6.85	96.1

*No IE, No Intestinal Extract; with IE, with Intestinal Extract.

**Based on the measured concentrations in the test metal(loid) samples in the presence and absence of intestinal extract.

Ethylene diamine tetraacetic acid (EDTA) and 2,3-dimercapto-1-propanesulfonic acid (DMPS); Escherichia coli and Lactobacillus acidophilus.

**Table 2:** Percentage distribution of metal(loid)s during permeability test using Caco-2 cell technique.

Metal(loid) sources*	Chelating agents/Gut microbes	Total input (µg)**	Percentage of total metal(loid)		
			Apical	Membrane retention	Basolateral
As—No IE	Control	945	19.05	5.74	76.19
As—with IE	Control	860	32.33	7.29	60.00
	EDTA	868	34.56	15.32	47.35
	DMPS	865	41.50	18.38	38.96
	*E. coli*	872	35.09	13.30	50.11
	*L. acidophilus*	856	35.63	23.25	37.62
Cd—No IE	Control	606	69.14	15.59	17.33
Cd—with IE	Control	460	68.04	17.65	13.61
	EDTA	472	65.89	19.36	11.69
	DMPS	465	67.53	17.59	9.78
	*E. coli*	481	58.84	32.22	8.23
	*L. acidophilus*	470	55.53	35.11	6.89
Hg—No IE	Control	923	37.81	4.40	61.21
Hg—with IE	Control	635	43.46	13.31	42.68
	EDTA	647	43.43	15.46	34.16
	DMPS	653	49.92	17.15	31.09
	*E. coli*	640	42.97	21.56	34.69
	*L. acidophilus*	648	42.28	23.30	26.54
Pb—No IE	Control	796	83.92	15.45	3.45
Pb—with IE	Control	530	78.49	18.53	2.74
	EDTA	553	71.07	22.42	2.04
	DMPS	546	69.41	25.64	1.71
	*E. coli*	538	54.46	43.68	1.41
	*L. acidophilus*	546	51.65	45.24	1.25

*No IE, No Intestinal Extract; with IE, with Intestinal Extract.

** Based on the measured concentrations in the test metal(loid) samples in the presence and absence of intestinal extract.

Ethylene diamine tetraacetic acid (EDTA) and 2,3-dimercapto-1-propanesulfonic acid (DMPS); *Escherichia coli* and *Lactobacillus acidophilus.*

**Figure 3:**
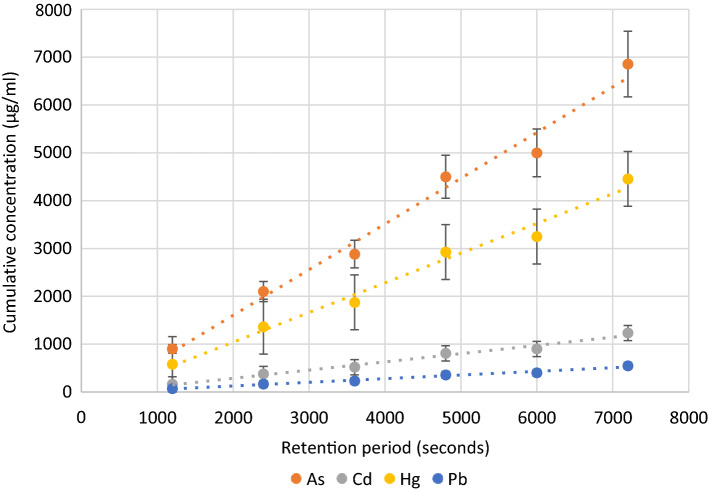
Time course of metal(loid) transport through Caco-2 cells for estimation of Apparent permeability (App) of metal(loid)s.

The time-course transport of MLs from the apical to basolateral compartment of Caco-2 monolayer is shown in Fig. 3. The amount of ML transported from apical to basolateral compartment increased linearly with time for all the MLs. The apparent permeability (*P*_app_) values of MLs were estimated using (Eq. 1) from the time-course of relationship ML transport shown in Fig. 3. The relative permeability values (*P*_rel_) were estimated using (Eq. 2) to examine the effect of various treatments (gut microbes and chelating agents) on intestinal apparent permeability values (*P*_app_).

**Figure 4:**
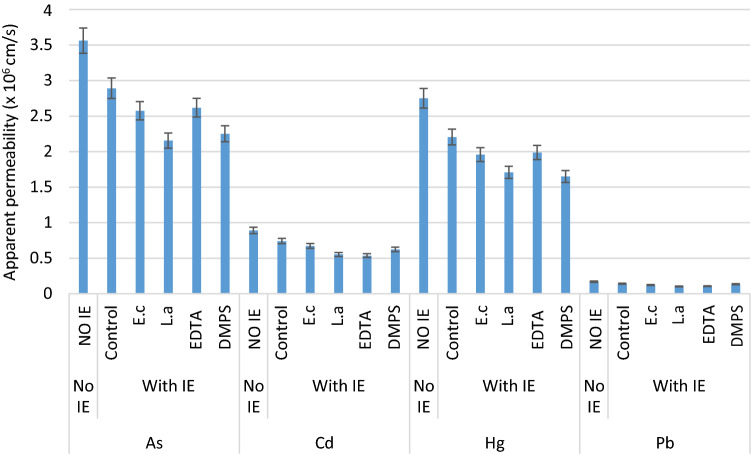
Effect of gut bacteria and chelating agents on the intestinal permeability of As, Cd, Hg and Pb. IE, intestinal extract; EDTA, ethylenediaminetetraacetic acid; DMPS, 2,3-dimercaptopropane-1-sulfonate; *E.c*, *Escherichia coli*; *L.a*, *Lactobacillus acidophilus.*

Addition of intestinal extract slightly decreased the transport of MLs from apical to basolateral compartment while increasing their cellular retention (Table 2; Fig. 4). The apparent permeability coefficient (*P*_app_) evaluates the velocity with which a solute crosses the cell monolayer. The *P*_app_ values for As, Cd, Hg and Pb were decreased by 7.5%, 6.3%, 7.9% and 8.2% in the presence of intestinal extract indicating less ML permeability. The *P*_app_ values varied between the MLs, and followed: As(III) > Hg(II) > Cd(II) > Pb(II).

### Effect of gut microbes in permeability of metal(loid)s

**Figure 5:**
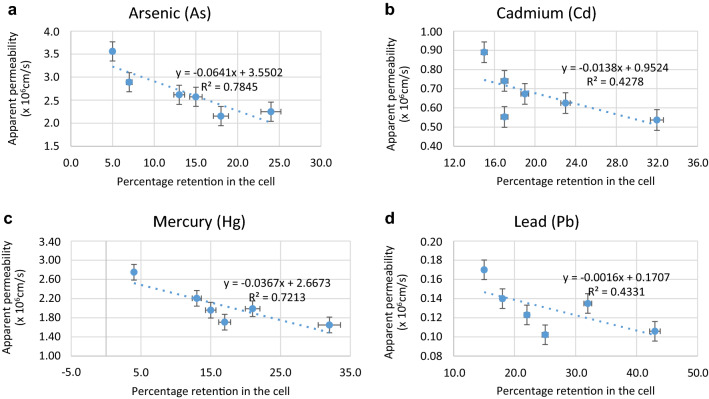
Relationships between percentage retention in the cell membrane and intestinal permeability of As, Cd, Hg and Pb.

**Figure 6:**
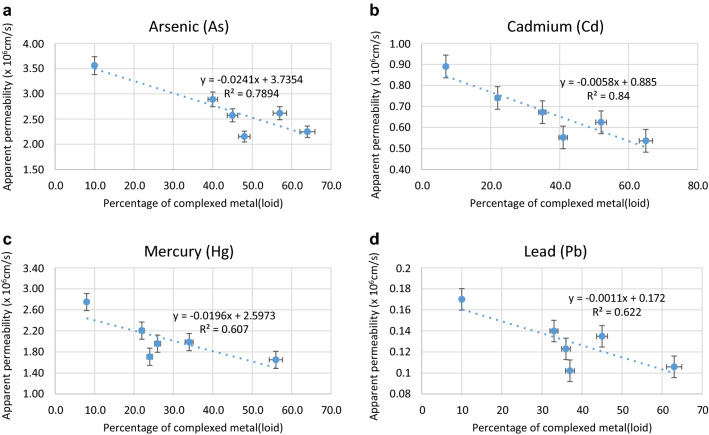
Relationships between percentage complexed metals and intestinal permeability of As, Cd, Hg and Pb.

Treatment with gut microbes significantly reduced the permeability of MLs in Caco-2 cells as seen from the relative permeability (*P*_rel_) values reported in Table 3. The apparent permeability (*P*_app_) values calculated from (Eq. 1) were markedly reduced in the presence of gut microbes for all the MLs indicating low intestinal absorption (Table 3; Fig. 4). The percentages of ML retained in the Caco-2 cell membrane and the ML complexed are presented in Table 3. There were significant positive relationships between the apparent permeability (*P*_app_) values and the amount of MLs retained in the Caco-2 epithelial cells (Fig. 5) and the amount of ML complexed (Fig. 6).

**Table 3:** Absolute and relative Apparent Permeability of metal(loid)s as measured by Caco-2 cell technique and percentage of metal(loid)s complexed.

Metal(loid) sources*	Chelating agents/Gut bacteria	Absolute App Permeability (× 10^–6^ cm/s)**	Relative App Permeability***	% Complexed metal(loid)s
As—No IE	Control	3.5600	100	10.2
As—with IE	Control	2.8907	81.2	40.4
	EDTA	2.5739	72.3	45.2
	DMPS	2.1542	60.5	48.1
	*E. coli*	2.6166	73.5	57.6
	*L. acidophilus*	2.2855	64.2	63.2
Cd—No IE	Control	0.8900	100	7.03
Cd—with IE	Control	0.7405	83.2	22.4
	EDTA	0.6728	75.6	35.3
	DMPS	0.5527	62.1	41.4
	*E. coli*	0.5367	60.3	65.4
	*L. acidophilus*	0.4931	55.4	68.7
Hg—No IE	Control	2.7500	100	8.01
Hg—with IE	Control	2.2055	80.2	22.2
	EDTA	1.9580	71.2	26.1
	DMPS	1.7078	62.1	24.3
	*E. coli*	1.9883	72.3	34.3
	*L. acidophilus*	1.7133	62.3	37.6
Pb—No IE	Control	0.1700	100	10.2
Pb—with IE	Control	0.1399	82.3	33.2
	EDTA	0.1229	72.3	36.4
	DMPS	0.1022	60.1	37.3
	*E. coli*	0.1059	62.3	63.3
	*L. acidophilus*	0.0904	53.2	65.6

*NO IE, No Intestinal Extract; with IE, with Intestinal Extract; **Absolute Apparent permeability is calculated from Eq. (1) (× 10^–6^ cm/s); ***Relative Apparent permeability is calculated from Eq. (2); absolute permeability for metal(loid) alone (No IE) is taken as 100%

Ethylene diamine tetraacetic acid (EDTA) and 2,3-dimercapto-1-propanesulfonic acid (DMPS); *Escherichia coli* and *Lactobacillus acidophilus.*

The effect of gut microbes on *P*_app_ varied both between the gut bacteria and also amongst the MLs. The adsorption of MLs by gut microbes was found to be in the order of Pb > Cd > Hg > As. In the presence of *L. acidophilus and E. coli*, the transport of MLs to the basolateral compartment decreased from 60.0 to 37.6% and 50.1% for As, from 13.6 to 6.89% and 8.23% for Cd, from 42.6 to 26.5% and 34.7% for Hg, and from 2.74 to 1.25% and 1.41% for Pb, respectively (Table 2). Correspondingly, the cellular retention of MLs was higher in the presence of gut microbes (Table 2).

### Effect of chelating agents on permeability of metal(loid)s

The results showed a significant reduction in the ML permeability in the presence of chelating agents (Table 3). The *P*_app_ values were lower in the presence of chelants indicating low intestinal absorption. However, the effect of chelants on the decrease in permeability of heavy MLs depended on the nature of MLs. While it was found that EDTA formed complexes with Cd and Pb more readily, thereby decreasing the permeability of MLs, DMPS readily formed complexes with As and Hg. In the presence of EDTA and DMPS, the transport of MLs to the basolateral compartment decreased from 60.0 to 47.3% and 38.9% for As, from 13.6 to 11.7% and 9.8% for Cd, from 42.6 to 34.1% and 31.1% for Hg, and from 2.74 to 2.04% and 1.71% for Pb, respectively (Table 3). Correspondingly, the cellular retention of MLs were higher in the presence of chelants (Table 3).

## Discussion

### Transport and apparent permeability

The apparent permeability (*P*_app_) values of MLs as measured by Caco-2 cell model using (Eq. 1) are presented in Table 3 and Fig. 4. The correlation between the absorbed fraction in humans (in vivo) and permeability across the Caco-2 monolayer (*P*_app_) (in vitro) has been evaluated in many studies^13,41–44^. Yee^44^ suggests that a drug compound with *P*_app_ < 1 × 10^–6^ cm/s shows low absorption in vivo (0–20%), while a *P*_app_ of between 1 and 10 × 10^–6^ cm/s indicates moderate absorption (20–70%), and *P*_app_ > 10 × 10^–6^ cm/s suggests high absorption (70–100%).

Calatayud et al.^45^ found a linear increase of As transport in Caco-2 cells with increasing input concentration (1 μM—67 μM), which suggests no saturable component in the transport within the concentration range tested in their study. The *P*_app_ values for As(III) and As(V) at 2 h for a concentration of 67 μM was 4.6 ± 0.3 × 10^–6^ cm/s and 1.00 ± 0.05 × 10^–6^ cm/s, respectively^45,46^. This indicates that As(III) species is more readily permeable through intestinal epithelial cells than As(V), which may contribute to higher toxicity of the former species to biota^47^. However, Laparra et al.^48,49^ noticed a decrease in *P*_app_ value when the As(III) concentration in the donor compartment was increased suggesting the existence of a saturable intestinal transport system for As(III). The *P*_app_ value was 1.1 ± 0.8 × 10^–6^ cm/s after 2 h of incubation at a concentration of 67 μM which was lower when compared to Calatayud et al.^45^. Similarly, Liu et al.^47^ observed lower *P*_app_ values for As(V) (4.6 ± 0.2 × 10^–7^ cm/s) and As(III) (1.6 ± 0.1 × 10^–6^ cm/s) after 2 h of incubation at a concentration of 3 μM. Variations in apparent permeability coefficients amongst various studies were attributed to the differences in transport medium and cell conditions (e.g., culture conditions, passage).

The transport and absorption of Cd across Caco-2 monolayers in combination with the Ussing chamber technique was investigated by Schar et al.^50^. They have demonstrated that the exposure of Caco-2 cells to different Cd concentrations caused a reduction of the proportion of Cd accumulation in cells from 38% (at 1 μM) to 13% (at 10 μM) indicating saturation of Cd binding sites at the outer apical or basolateral membrane. An earlier in vivo study by Foulkes^51^ showed a saturation of Cd-binding sites in the rat jejunum. The Cd transport across the Caco-2 monolayers in the present study was linear (Fig. 3). This is in agreement with a study on Cd transport across Caco-2 cells by Blais et al.^52^. They found that Cd transport into the basolateral compartment was much slower and was undetectable during a lag time of about 60 min indicating a linear transport. This also suggests that Cd uses the cellular or carrier pathway to move across the intestinal epithelium. In addition, after 24 h only a small part of the Cd accumulated in the Caco-2 cells (6 to 12%) and the remainder was found in the basolateral compartment.

In an in vitro digestion/Caco-2 cell model study, Chunhabundit et al.^53^ found that the cellular Cd uptake of inorganic Cd from CdCl_2_ solution was significantly higher than that of the soluble Cd from food (pig kidney/kale) or CdCl_2_ digests. Earlier studies reported that 25% of Cd was taken up (both retained in the cells and transferred through cells) by Caco-2 cells from CdCl_2_ solution, while only 4–16% and 3.8–6.3% of Cd were taken up from leafy vegetables and infant food^54–56^. The lower Cd uptake from food suggests that the interaction or exchange between Cd and ligands in each food digest affect the intestinal Cd uptake.

In the current study, the lowest apparent permeability values were obtained for Pb (Table 3; Fig. 4). Fu and Cui et al.^56^ used a Caco-2 cell model to study the bioaccessibility and bioavailability of Pb in raw/cooked pakchoi (*Brassica rapa* L.) and Malabar spinach (*Basella rubra* L.). After incubation for four hours, they observed 9.4% Pb bioavailability in raw vegetables, against 3.2% in cooked vegetables. Further, they observed that raw spinach showed higher (four times) Pb bioavailability while it was two times in raw pakchoi. Overall, the Pb bioavailability ranged from 2.0 to 13.0% for the leafy vegetables.

There are several factors that affect the bioavailability of MLs such as food constituents, digested products, selection of assays (in vivo*/*in vitro), and the incubation for the chosen cell culture assay (e.g. Caco-2 cells). For instance, Yannai and Sachs^57^ observed 1.4% and 0.9% of fish meal Pb in kidney and liver, respectively. Similarly, another study^58^ using Pb from mine waste and Pb acetate measured Pb concentration in blood and estimated the absolute bioavailability values to be 15% and 2.7%, respectively. For Caco-2 cells, 30% of Pb was absorbed (Pb associated and transported by Caco-2 cells) from the digested soil solution^14^. After 24 h, the cells retained approximately 27% of Pb while the cells moved 3% of Pb through the single layer, and a transcellular pathway was considered as the main mechanism of transport across the epithelial layer. Furthermore, since the free Pb^2+^ concentration in small intestinal fluid/chyme was negligible, results revealed the contribution of Pb phosphate and Pb bile complexes in chyme to the Pb flux towards the cells^14^.

Vázquez et al.^59^ evaluated the accumulation and transport of Hg(II) using Caco-2 cells as an intestinal epithelium model. The *P*_app_ values for Hg(II) after 120 min of exposure increased with increasing concentration tested, though the increase was only significant for the 1 mg/L concentration (*P*_app_ 0.1 mg/L = 1 ± 0.13 × 10^–6^ cm/s; 0.5 mg/L = 1.4 ± 0.5 × 10^–6^ cm/s; 1 mg/L = 3.8 ± 0.32 × 10^–6^ cm/s). The ML showed moderate absorption, and its transport fundamentally took place via a carrier-mediated transcellular mechanism. A major observation was that the cellular accumulation of Hg(II) (21–51%) from the initial addition to the apical media was far greater than the transport to the basolateral side (9–20%). A similar observation of cell retention of Hg was found in the present study. Vázquez et al.^59^ noted that the in vivo studies using Hg(II) exhibits an absorption of < 15%, which is lower than that deduced from the assays using Caco-2 cell line.

While few researchers^45,60,61^ also observed increased cellular uptake of Hg added as a pure ML solution, the presence of luminal factors (e.g. bile salts, food components) reduces Hg transport across the intestinal epithelial cells as in the case of in vivo studies^62^. Similarly, in a Caco-2 cell model, Calatayud et al.^45^ found a higher cell retention (49–69%) and a much lower transport of bioaccessible fraction of swordfish Hg to the basal compartment (3–14%) after 2 and 4 h. In a study by Vázquez et al.^62^, the components solubilised during gastrointestinal digestion of swordfish reduced the entry of CH_3_Hg into Caco-2 monocultures and hence, resulted in reduced cellular accumulation. They demonstrated that in the case of inorganic HgCl_2_ standard prepared in the gastrointestinal digestion blank, the presence of food matrix significantly increased the non-absorbed percentage (from 55% to ≥ 73%) and greatly reduced cell uptake (from 33 to 11%) during a period of 60 min.

Overall, the results in the present study demonstrated lower ML transport in the presence of intestinal extracts, which is related to some complexing components such as bile salts in the intestinal solution. These complexing components can also affect ML absorption because of competition for transport or due to the formation of complexes with ML, which has a lower transport rate^63^. High retention of MLs in Caco-2 cells indicate that the intestinal epithelium acts as a barrier for ML absorption. The apparent permeability of MLs was in the order of: As(III) > Hg(II) > Cd(II) > Pb(II). While the anionic As transport can be passive and fast, the transport of remaining MLs which are cations, mostly occur by active transport, and hence can be slower than As.

### Effect of gut microbes in permeability of metal(loid)s

The ability of gut bacteria to adhere to mucus and/or intestinal epithelial cells is one of the major mechanisms protecting the host from contaminant invasion and adhesion^64^. The effect is observed even if the bacterial adhesion is transient and does not lead to permanent intestinal colonisation^65–67^. One of the major objectives in this study was to determine the amount of ML transport across the Caco-2 cell monolayer in the presence of gut bacteria. The positive relationships between apparent permeability, and ML retention and permeability (Figs. 5 and 6) indicates that metal(loids) retained by the epithelial cells may not be transported across the cells, and also only free ML species are transported across the cells^63,68^. Therefore, the results observed in this study may be attributed to a direct protection of the intestinal barrier against the MLs or indirectly via intestinal ML sequestration by the gut microbes^69,70^.

Using Caco-2 cells, Monachese et al.^71^ compared the amount of Pb and Cd in the basolateral chamber in non-treated wells to *Lactobacilli* pre-treated wells and noticed a significant reduction (50% and 90% reduction in Pb and Cd, respectively) in measured MLs after a period of 5 h when pre-treated. This observation greatly supports ML binding by *Lactobacilli* and reduced absorption by the Caco-2 cell line. Muhammad et al.^72^ recently demonstrated a notable Pb binding capacity and tolerance capability of *L. plantarum* KLDS 1.0344. Oral administration of both free and encapsulated KLDS 1.0344 significantly provided protection against induced chronic Pb toxicity by increasing faecal Pb levels and by decreasing blood Pb levels in mice.

Caco-2 cell cultures have been widely used to investigate the adhesion of various gut microorganisms including *Lactobacillus* strains to epithelial cells^73–75^. The gut microbes adhere to human intestinal cells via mechanisms, which involve different combinations of carbohydrates and proteins on the bacterial cell surface^67,76^. The adhesion ability of gut microbes may differ in various cellular models used for examining the intestinal permeability of drugs, nutrients and metals. For example, Sarem et al.^77^ noticed varying degrees of *Lactobacillus* strain adhesion in two cellular models – human epithelial intestinal Caco-2 and Int-407 cell lines. Depending on the origin and the dose, the gut bacteria represent different adhesive properties^78,79^. For instance, while one study reported *L. rhamnosus* as a strain with low ability to adhere to the epithelial cells, few other studies indicated the adhesive properties of *L. rhamnosus* in the range of 7.2–14.4%^80^ and at the level of 20%^81,82^.

Exposure to contaminants including MLs is associated with an increase in gut permeability, leading to ‘leaky gut syndrome’^26,27^. Exposure to Cd, for instance, causes significant damage to the gut barrier, including the toxicity of enterocytes, induction of inflammatory response, and disruption of tight junctions, as demonstrated by^16^. However, gut bacteria can help in the modulation of contaminants-induced leaky gut syndrome through their effect on sequestering contaminants such as heavy MLs^83,84^. For example, *L. plantarum* strains markedly decreased the permeability of Cd, thereby mitigating the Cd-induced leaky gut syndrome^16^. In their study, a clear protection against damage of HT-29 cells was observed when *L. plantarum* CCFM8610 gut bacteria was introduced simultaneously with Cd exposure (intervention assay) which they partly attributed to the intestinal Cd sequestration by gut bacteria thereby attenuating Cd exposure. Treatment with CCFM8610 significantly alleviated Cd-induced cytotoxicity and reversed the disruption of tight junctions in HT-29 cells. They further confirmed that the bacteria can inhibit Cd absorption by protecting the intestinal barrier in Cd-exposed mice. The presence of *Lactobacillus* sp. demonstrated significantly increased faecal Cd levels and decreased Cd accumulation in the tissues of Cd-exposed mice, and also a notable decrease in the intestinal permeability of Cd. This suggests that modulating the gut microbiota can serve as a potential strategy for regulating intestinal permeability and may help to alter the course of autoimmune diseases in susceptible individuals^27,85^.

Gut bacteria have been shown to adsorb MLs including As(III), As(V), Cd(II), Pb(II) and Hg(II), and the extent of adsorption varied between the MLs and gut bacteria, which is attributed mainly to difference in the nature of functional groups between the bacteria. The reduction in the bioaccessibility of MLs in various sources by gut microbes could be attributed to the immobilisation through adsorption, complexation, and precipitation reactions^86^. The microbial cell wall is a natural barrier for MLs, since the functional groups of several macromolecules are involved in the immobilisation of MLs. In Gram-negative bacteria, lipopolysaccharide, a major component of the outer membrane, is effective in the immobilisation of ML ions. In Gram-positive bacteria, peptidoglycan along with teichoic and teichuronic acids are involved in ML binding.

The positive relationships between apparent permeability, and metalloid retention and permeability (Figs. 5 & 6) indicates that metal(loids) retained by the epithelial cells may not be transported across the cells, and also only free ML species are transported across the cells^63,68^.

### Effect of chelating agents on permeability of metal(loid)s

Chelating agents have been shown to increase in the bioaccessibility of MLs, which is attributed to the complexation/chelation of MLs by the chelating agents and the subsequent increase in the solubilisation of these MLs from the respective ML sources. However, the two chelating agents in this study have been found to decrease the bioavailability of MLs as measured by intestinal permeability test. Dietary fibres, thiol-containing compounds such as cysteine, homocysteine, albumin and glutathione, and phytochemicals present in natural foods such as tea, can also act as chelants in lowering the intestinal absorption of contaminants including heavy MLs^87–92^. One of the mechanisms for reducing ML permeability is the formation of soluble ML complexes whose transport is less than that of the free forms of the ML species^93^. There were significant negative relationships between the apparent permeability (*P*_app_) values and the amount of MLs complexed (Fig. 6). The effect of dietary compounds on the transport of Hg present in the bioaccessible fraction (CH_3_Hg) of swordfish was examined by Jadán-Piedra et al.^94^ using a Caco-2 model. The *P*_app_ values of Hg in the presence of cysteine and homocysteine were reduced by 38% and 35%, respectively. Similarly, Vázquez et al.^60,61^ showed a decrease in the cellular accumulation of CH_3_Hg by up to 55% in the presence of cysteine derivatives via Caco-2 cell model.

Clemente et al.^95^ examined the influence of dietary compounds including phytochemicals such as cysteine and glutathione, on the bioavailability of As(III) as measured by intestinal permeability using colon-derived human cells (NCM460 and HT-29MTX). Their findings demonstrated significant decreases in the quantity of As(III) transported across the epithelial monolayer in the presence of dietary compounds with a marked decrease in the presence of cysteine. The permeability of As(III) was reduced by 70%, 59%, 45% and 44% by cysteine, glutathione, epicatechin and homocysteine, respectively. The *P*_app_ values were decreased by 63% in the presence of cysteine. The binding of inorganic As to sulfhydryl groups is considered as one of the main mechanisms for As toxicity because its binding to a protein through cysteine residues alters the conformation and function of the protein^96^. Also, the complexes formed between As(III) and free cysteine are insoluble in the pH range of 4−8 which partially explains the decrease in the transport of inorganic As across the intestinal cell monolayer in the presence of cysteine^97^.

## Conclusions

The ability of a ML to pass through the gastrointestinal barrier is an essential process when investigating the bioavailability and toxicity of heavy MLs. The process of absorption may be impacted by the ML binding with gut microbes or by competition with compounds that reduce its solubility or its passage through the epithelium. This study demonstrated that gut microbes and chelating agents could decrease in the permeability of MLs. Chelating agents reduce intestinal absorption of MLs by forming complexes thereby making them less permeable. Whereas in the case of gut bacteria, the decrease in the intestinal permeability of MLs may be associated to a direct protection of the intestinal barrier against the MLs or indirect intestinal ML sequestration by the gut bacteria through adsorption. Thus, both gut microbes and chelating agents can be used to decrease the intestinal permeability of heavy MLs, thereby mitigating their toxicity.

## Supplementary Information

Supplementary Tables.
