# Supplementary material for: Bioavailability of arsenic, cadmium, lead and mercury as measured by intestinal permeability
Source: Sci Rep. 2021 Jul 19;11:14675. doi: 10.1038/s41598-021-94174-9 (PMC8289861; doi:10.1038/s41598-021-94174-9)
Supplement: Supplementary file 1 — Supplementary Tables. [file 41598_2021_94174_MOESM1_ESM.docx]

Bioavailability of arsenic, cadmium, lead and mercury as measured by intestinal permeability

Shiv Bolan^1,2^, Balaji Seshadri^1,2^, Simon Keely^3^, Anitha Kunhikrishnan^1^, Jessica Bruce^3^, Ian Grainge^4^, Nicholas J. Talley^3^ and Ravi Naidu*^1,2^

^1^Global Centre for Environmental Remediation, University of Newcastle, NSW, Australia.

^2^Cooperative Research Centre for Contamination Assessment and Remediation of the Environment, University of Newcastle, NSW, Australia

^3^Hunter Medical Research Institute, University of Newcastle, NSW, Australia

^4^School of Environmental and Life Sciences, University of Newcastle, NSW, Australia

*Corresponding author: [Ravi.Naidu@newcastle.edu.au](mailto:Ravi.Naidu@newcastle.edu.au)

**Supplementary Tables:**

Supplementary Table 1. Bacterial species used

Supplementary Table 2. Chelating agents used in this study

Supplementary Table 1. Bacterial species used

| **Bacteria** | **Family** | **Primary location** | **Optimum pH** | **Cell wall** |
| --- | --- | --- | --- | --- |
| *Lactobacillus acidophilus* | Lactobacillaceae | Mouth and stomach | 3 - 5 | Gram positive |
| *Escherichia coli* | Enterobacteriaceae | Large intestine (lower) | 6 - 7.5 | Gram negative |

Supplementary Table 2. Chelating agents used in this study

| **Chelating agent** | **Properties** | **Metal(loid)s complexed** | **Treatment delivery** | **Chelation dose** |
| --- | --- | --- | --- | --- |
| EDTA | C_10_H_16_N_2_O_8_  292.24 g·mol^−1^  0.860 g cm^−3^ (at 20 °C)  aminopolycarboxylic acid; colorless and water-soluble solid | Cd, Pb, Hg, As, Cu, Zn | Mainly Intravenous; sometimes oral | 1.2–2 g/d |
| DMPS | C_3_H_8_O_3_S_3_  M.wt; 188.289g/mol  Water soluble | Hg, As, Cd, Pb | Mainly oral; sometimes intravenous | 10–30 mg/kg/d |

*EDTA = Ethylene diamine tetra acetic acid; DMPS = 2,3-dimercaptopropane-1-sulfonate; DMSA = 2,3-dimercaptosuccinic acid*
